# Changes in substance P levels of inferior turbinate in patients with mucosal contact headache^☆^^☆☆^

**DOI:** 10.1016/j.bjorl.2019.01.006

**Published:** 2019-02-22

**Authors:** Hülya Eyigör, Mete Eyigör, Bekir Erol, Ömer Tarık Selçuk, Levent Renda, Mustafa Deniz Yılmaz, Üstün Osma, Cansu Demirkıran, Meral Gültekin, Nuray Erin

**Affiliations:** a Antalya Education and Research Hospital, Department of Ear-Nose-Throat Head and Neck Surgery, Antalya, Turkey; b Akdeniz University Medical Faculty, Department of Medical Microbiology, Antalya, Turkey; c Antalya Education and Research Hospital, Department of Radiology, Antalya, Turkey; d Akdeniz University Medical Faculty, Department of Medical Pharmacology, Antalya, Turkey

**Keywords:** Substance P, Inferior turbinate hypertrophy, Contact point headache, Visual analog scale, SNOT-22, EIA, Substância P, Hipertrofia das conchas inferiores, Cefaleia de ponto de contato, Escala analógica visual, SNOT-22, EIA

## Abstract

**Introduction:**

Mucosal contact headache is a referred pain that arises from contact between the nasal septum and the lateral nasal wall. Evidence supports the role of substance P in a contact headache such that release of substance P from sensory nerve endings causes inflammation and allergy.

**Objectives:**

This study aimed to determine possible differences in substance P levels in inferior turbinate hypertrophy creating a contact headache.

**Methods:**

28 patients who had contact headaches (study group) and 16 volunteers with no complaints were included in the study. Substance P levels in the inferior turbinate tissue samples were quantified using a commercially available substance P EIA kit.

**Results:**

In the study group average substance P levels were 2.65 ± 0.27 pg/mg tissue (range: 0.61–5.44) and in the control group it was 1.77 ± 0.27 pg/mg tissue (range: 0.11–4.35). The difference was statistically significant between the two groups (*p* = 0.0215). Average preoperative headache group visual analog scale scores was 5.93 ± 0.38 (2–9) and the turbinate volume was 6.56 ± 0.35 cm^3^ (3.50–10.30). The control group turbinate volume was 4.71 ± 0.39 cm^3^ (2.50–7.70). We found a correlation between the visual analog scale scores and substance P levels such that substance P levels were higher in visual analog scale scores above 5 (*p* = 0.001).

**Conclusion:**

This study demonstrates the relationship between intranasal contact headaches and increased mucosal substance P levels. We also found that there is no correlation with substance P levels and volume of the inferior turbinate.

## Resumo

**Introdução:**

A cefaleia por ponto de contato da mucosa é uma dor direcionada que surge do contato entre o septo nasal e a parede nasal lateral. Evidências corroboram o papel da substância P na cefaleia de contato, de tal forma que a liberação da mesma a partir de terminações nervosas sensoriais possa causar inflamação e alergia.

**Objetivo:**

Determinar possíveis diferenças nos níveis da substância P na hipertrofia de conchas inferiores em relação à cefaleia de contato.

**Método:**

Foram incluídos no estudo 28 pacientes que apresentaram cefaleia por ponto de contato (Grupo Estudo) e 16 voluntários sem queixas. Os níveis de substância P nas amostras de tecido da concha inferior foram quantificados com um kit substância P EIA, comercialmente disponível.

**Resultados:**

No grupo do estudo, os níveis médios de substância P foram 2,65 ± 0,27 pg/mg de tecido (variação: 0,61–5,44) e no grupo controle foram de 1,77 ± 0,27 pg/mg de tecido (variação: 0,11-4,35) e a diferença foi estatisticamente significante entre os dois grupos (*p* = 0,0215). O escore médio da escala visual analógica do grupo de cefaleia pré-operatória foi de 5,93 ± 0,38 (2–9) e o volume das conchas foi de 6,56 ± 0,35 cm^3^ (3,50–10,30). O volume da concha do grupo controle foi de 4,71 ± 0,39 cm^3^ (2,50 ± 7,70). Encontramos uma correlação entre o escore da escala visual analógica e os níveis de substância P, de modo que os níveis de substância P foram maiores nos escores da escala visual analógica acima de 5 (*p* = 0,001).

**Conclusão:**

Este estudo demonstra a relação entre cefaleias por contato intranasais e níveis aumentados de substância P nas mucosas. Também observamos que não há correlação com os níveis de substância P e o volume da concha inferior.

## Introduction

Mucosal contact headache is a referred pain that originates from contact between the nasal septum and the lateral nasal wall which might be due to nasal septal deviations, septal spurs and concha bullosa of middle turbinate.^1^ Computed tomography (CT) and nasal endoscopy are often used to evaluate patients with suspected mucosal contact headache.^2^ A contact point headache is diagnosed based on four criteria revised in 2013 by the Headache Classification Committee.^3^ These criteria are: (1) intermittent pain located in the periorbital or medial canthus, and temporozygomatic regions; (2) headache that disappears within 5 min following topical application of topical anesthesia to the contact point area; (3) confirmed presence of mucosal contact point by both, nasal endoscopy and sinus CT scan, without the presence of acute or chronic rhinosinusitis, nasal polyps, and nasal cavity tumors; (4) headache disappears within 7 days after resection of mucosal contact point.

Although Stammberger and Wolf had previously hypothesized that neuropeptides, especially the substance P (SP), are involved in the mediation of facial pain due to mucosal contact points, there is a limited number of studies examining the changes in Substance P levels in this group of patients.^4^

Indirect evidence also supports the role of SP in contact headache in that release of SP from sensory nerve endings causes inflammation and allergy.^2^, ^4^ Specifically, the local release of Substance P due to allergens or irritation causes vasodilatation and hypersecretion, which may lead to hypertrophic changes.^4^, ^5^

Substance P which acts by binding to the NK-1 receptor (NK-1R) is not only an acute inflammatory mediator but also considered as a pain mediator.^6^ Unmyelinated sensory nerve fibers of mucosa of nasal cavity include SP which may be released during mucosal contact and induce referred pain.^4^ SP-containing nerve fibers in the human inferior turbinate are mainly found in the walls of arterioles, venules, sinusoids, gland acini, near the basement membrane. SP-binding sites were also demonstrated in arterioles, venules, and glands.^7^ Recently, the presence of SP and NK-1R was shown by immunohistochemistry and RT-PCR demonstrating its role in a contact headache.^8^ To our knowledge, the actual level of SP in inferior turbinate hypertrophy and its correlation with inferior turbinate volume has not been studied before. Hence in the present study, we examined the SP levels in mucosal samples obtained from inferior turbinate hypertrophy in patients with and without contact headache.

## Methods

### Study subjects and design

The study is conducted according to the Helsinki Declaration and was approved by the Ethics Committee of Antalya Education and Research Hospital (Date: 12.02.2015; no. 54/10). Informed consents were obtained from all subjects.

Patients (*n* = 28) who applied to Antalya Education and Research Hospital Ear-Nose-Throat (ENT) clinic because of complaints of headache and had endoscopic and CT confirmed inferior turbinate hypertrophy with septo-conchal contact were included in the study. These patients had turbinate reduction surgery (Study Group). The control group consisted of 16 patients who had septum surgery and/or turbinate reduction without a contact-type headache (Control Group).

The inclusion criteria and exclusion criteria were the same as those previously reported (Headache Classification Committee).

Individually intranasal mucosal contact headache was considered positive when at least 2 of the criteria determined by Headache Classification Committee were present.^3^ Patients who had hypertrophy of the turbinate inferior and did not respond to the medical treatment for at least three months and responded to lidocaine were selected for the surgical procedure.^9^ Pain score was determined by using a preoperative Visual Analog Scale (VAS). VAS 0 was defined as “no headache” and ten as “severe pain”.^10^ All patients completed the SNOT-22 questionnaire and were scored by ENT specialists.

### Assessing the volume of the turbinate in Paranasal sinus CT

**Figure 1:**
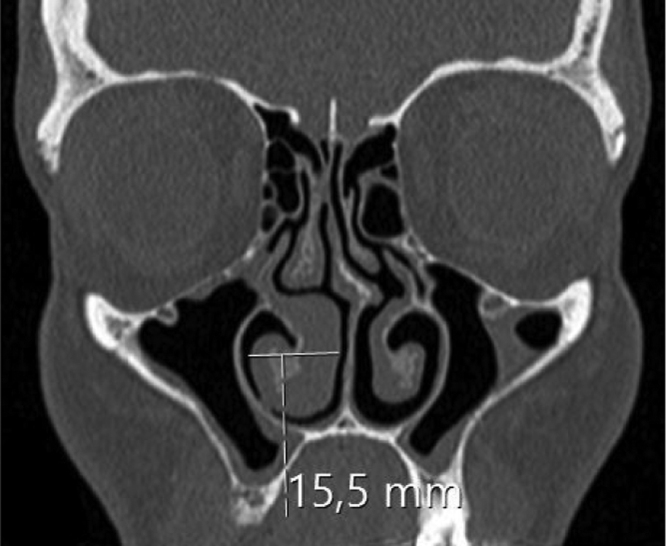
Right hypertrophic inferior concha and measurements of width are seen.

**Figure 2:**
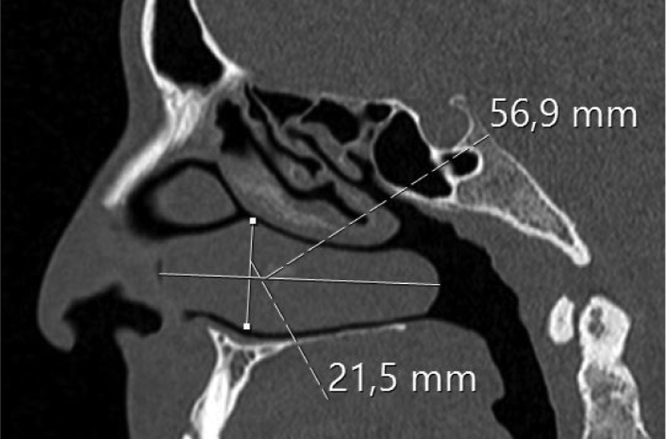
Right hypertrophic inferior concha and measurements of length and height are seen.

The inferior turbinate size was measured with coronal and sagittal reformatted CT images. Concha width (*A*) from Coronal images and concha length (*B*) and height (*C*) from sagittal images were recorded by measuring the widest point. Inferior turbinate volume was calculated using the 3D volume formula (*A* × *B* × *C* × 0.52) (Figure 1, Figure 2).

### Samples and measurements of Substance P

**Figure 3:**
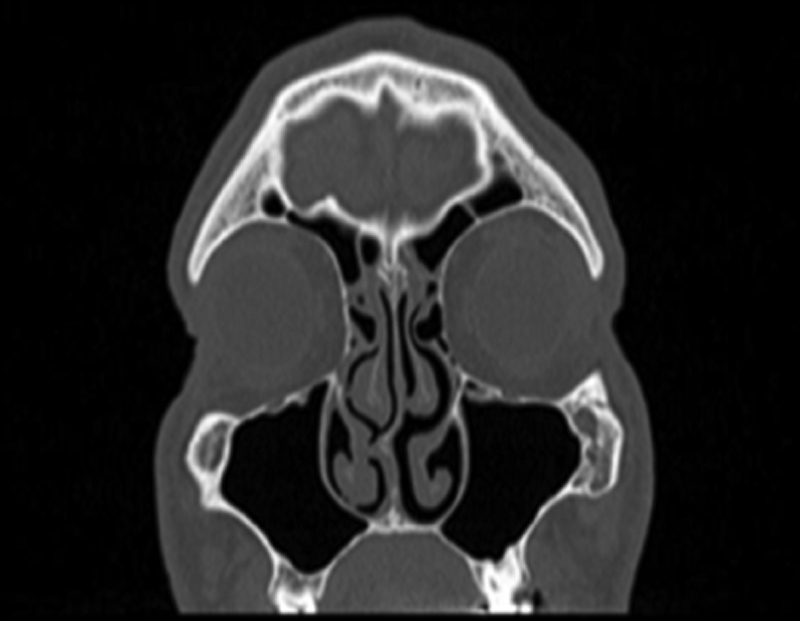
Septo-conchal mucosal contact is seen.

Conventional submucosal turbinoplasty or microdebrider-assisted inferior turbinoplasty was performed in all patients. Surgical specimens were taken from the inferior concha surface that had contact with the septum in patients, and were taken from the medial surface of inferior turbinate in the control group (Fig. 3). We previously established a technique to quantify SP levels mainly found in C-fibers and extractions were performed accordingly.^11^ Specifically 10-min acetic acid extraction was used to extract SP levels in neuronal tissues. SP levels than were quantified with an SP EIA kit (Cayman Chemical, USA) according to the manufacturer's instructions.

### Statistical analysis

All statistical calculations were done using Instat Sofware. Results were given as mean ± standard error. Statistical differences were analyzed by Student's *t*-test. The statistical significance level was established at *p* < 0.05.

## Results

**Figure 4:**
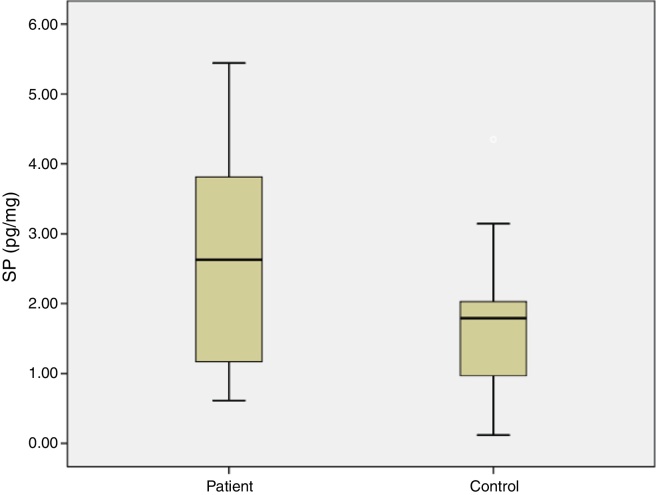
Substance P levels in groups.

**Figure 5:**
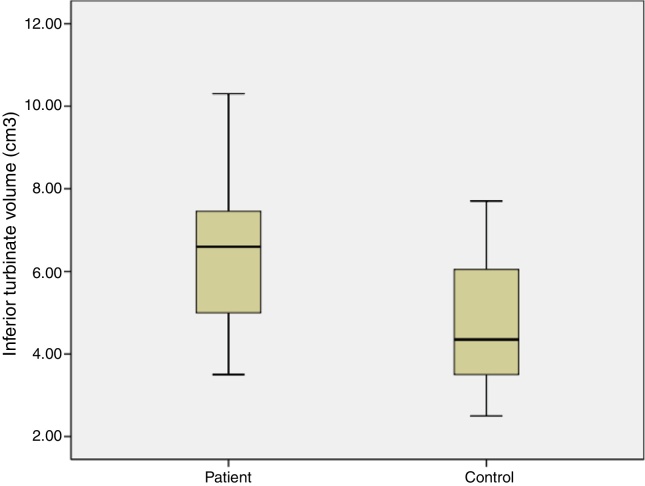
Inferior turbinate volume in groups.

The average age of patients in the study group was 30.5 ± 2.04 (18–54), while the average age of patients in the Control Group was 31.9 ± 3.12 (18–63). The patient group consisted of 19 men, nine women and the control group consisted of 10 male and 6 women. Significant increases in SP levels were observed in the study group compared to the Control Group (*p* = 0.0215) (Fig. 4). Inferior turbinate average volumes of patients in the study group was 6.56 ± 0.35 (3.5–10.3 cm^3^) and 4.71 ± 0.39 in the Control Group (2.5–7.7) (Fig. 5). Inferior turbinate average volumes of patients in the study group were not significantly different (*p* > 0.05) (Table 1). VAS score decreased significantly after surgery. Specifically the VAS score of preoperative headache was 5.95 ± 2.03 (2–9), and it was 1.39 ± 0.92 (0–4) postoperatively (*p* = 0.00). The SNOT-22 score was similar among study and Control Group. SNOT score three months after surgery decreased markedly in both study and Control Group. The preoperative SNOT-22 questionnaire average scores were given in Table 1. The postoperative SNOT-22 score was 6.68 ± 1.49 (0–29) and 5.25 ± 1.95 (0–27) in the study and Control Groups respectively.

**Table 1:** Preoperative data of the patients.

	Patient (*n* = 28)	Control (*n* = 16)
Gender (M/F)	19/9	10/6
Age	30.5 ± 2.04 (18–54)	31.9 ± 3.12 (18–63)
Preoperative VAS	5.95 ± 0.39 (2–9)	–
Preoperative SNOT-22	24 ± 2.9 (5–69)	18.25 ± 3.42 (2–45)
SP levels (pg/mg)	2.65 ± 0.27 (0.61–5.44)	1.77 ± 0.27 (0.12–4.35)
Inferior turbinate volume (cm^3^)	6.56 ± 0.35 (3.5–10.3)	4.71 ± 0.39 (2.5–7.7)

**Figure 6:**
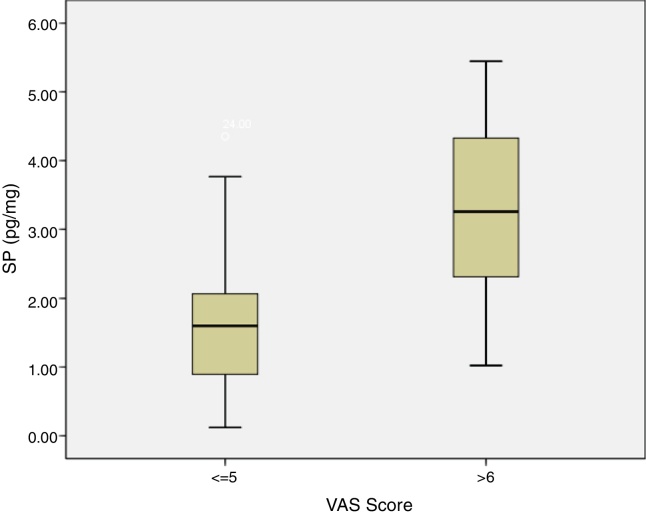
Correlation of VAS Score and SP levels in patients.

SP levels of the study group with VAS score of 5 and below were significantly lower compared to SP levels of the patients with VAS score of 6 and above (*p* = 0.0012) (Fig. 6). There was no statistically significant correlation between concha volume and SP values in both groups. Similarly, there was no statistically significant correlation between the SNOT-22 score and SP levels in both groups.

## Discussion

We here found that SP levels significantly increased in patients with a contact headache. Furthermore, there was a direct correlation between VAS score and SP levels in that patients with more severe headache had higher levels of SP in their hypertrophic concha. Recently Zhao et al. demonstrated the presence of SP, and it is receptor Neurokinin 1 (NK-1R) in intranasal mucosal contact points by immunohistochemistry. They found that mRNA level of SP and NK-1R was upregulated in nasal mucosa at contact points compared with non-contact points.^8^ To our knowledge, this is the first study demonstrating a correlation between headache and SP at protein levels.

Stammberger and Wolf reported that normal mucosa has higher concentrations of SP than chronic hyperplastic mucosa which seems to counteract to our findings.^4^ The difference, however, could be due to the extraction protocol used since SP is found both in neuronal and non-neuronal tissues.^12^ We mainly measured the neuronal SP content of the tissues. Alternatively, this might also be due to sampling of the concha such that we specifically obtained the tissues from contact points when available.

Several hypotheses were proposed regarding the pathogenesis of mucosal contact point headache. It was suggested that middle turbinate hypertrophy causes the loss of the gap between the nasal septum and the concha, resulting in mechanical compression of the ethmoidal nerves leading to headache.^12^ It was also speculated that mechanical stress on the nasal mucosal contact point activates sensory-C fibers leading to release of SP. SP, in turn, initiates headache.^4^ Our results supported the second hypothesis and further documented that actual SP levels were increased in contact headache patients.

SP belongs to the tachykinin family of peptides and is located mainly in sensory-C fibers which are widely distributed throughout the body.^6^ SP is also involved in the transmission of pain.^4^ SP-containing sensory-C fibers were documented in human nasal mucosa.^13^ The biological actions of SP, including pain transmission, is mainly mediated by NK1R.^14^ Nerve fibers displaying Substance P immunoreactivity have been detected in the nasal mucosa of several mammals.^7^

Abu-Bakra et al. applied SP topically to the nasal mucosa of 10 healthy subjects and found that SP caused nasal itching and sneezing but not headache; this results in questions regarding SP's role in headache.^13^ It is, however, possible that those patients with a contact headache may react to SP differently than healthy subjects and more work should be done in this group of patients. Furthermore, SP might be inducing pain indirectly by increasing swelling and local inflammation.^15^

Clear consensus regarding the therapeutic value of surgical correction of contact points in patients with a contact headache is not established. For example, Abu-Bakra et al. showed that surgical operation did not decrease facial pain.^16^ Zhao G. et al. reported persistence of headache in 5 of 40 patients with a contact headache after endoscopic surgery despite the presence of radiological absence of contact points, demonstrating that in a subgroup of patients headache is not only due to contact points.^8^ On the other hand, it was shown that a contact headache decreased 60%–89% after surgery.^17^ Similar findings were also reported by Mohebbi A et al. who demonstrated 83% decrease in contact headache after surgery.^5^

Furthermore Welge-Luessen's demonstrated long-term (10 years) protective effects of endonasal surgery in patients with a contact headache refractory to conservative therapy.^9^ Although in our study, the VAS score was significantly decreased after surgery, 3 of 28 patients had VAS score above or equal to 3, 3 months after surgery. Of these patients, 2 of them responded partially (VAS pre-operative value 7 and 5 – post-operative value 3 in both patients). These findings are in accordance with previous results discussed above and document that in a small percentage of a patient, factors other than mucosal contact are also responsible from a contact headache. Further studies are required to clarify other factors involved in resistance to surgical interventions.

Kaise et al. demonstrated that SP receptor (NK1R) antagonist inhibits the nasal obstruction induced by the antigen–antibody reaction in guinea pigs.^18^ SP also caused a dose-dependent increase in nasal airway resistance, an effect comparable to the effects of histamine, leukotriene D4 and antigen.^19^ NK1R antagonists are used clinically in the prevention of chemotherapy-induced emesis.^20^ These findings demonstrate that NK1R antagonists might inhibit allergic nasal obstruction and may also be useful in the treatment of a contact headache.

## Conclusion

We have demonstrated the relationship between intranasal contact headaches and increased mucosal SP levels. Furthermore, surgical intervention found to be effective in the majority of patients resistant to other conventional treatments. Our findings together with previously published data suggest that inhibition of SP activity with an NK1R antagonist may relieve the symptoms in a contact headache and may prevent hypertrophic changes. Further studies are needed to explore these possibilities.

## Conflicts of interest

The authors declare no conflicts of interest.
